# The effects of silver nitrate on *Mycobacterium abscessus* biofilms in a simulated antimicrobial showerhead environment

**DOI:** 10.3389/fpubh.2025.1572869

**Published:** 2025-05-26

**Authors:** Sarah Pitell, Cheolwoon Woo, Jill Millstone, Janet Stout, Leanne Gilbertson, Sarah-Jane Haig

**Affiliations:** ^1^Department of Civil and Environmental Engineering, University of Pittsburgh, Pittsburgh, PA, United States; ^2^Department of Chemistry, University of Pittsburgh, Pittsburgh, PA, United States; ^3^Department of Mechanical Engineering and Materials Science, University of Pittsburgh, Pittsburgh, PA, United States; ^4^Department of Chemical and Petroleum Engineering, University of Pittsburgh, Pittsburgh, PA, United States; ^5^Special Pathogens Laboratory, Pittsburgh, PA, United States; ^6^Department of Civil and Environmental Engineering, Duke University, Durham, NC, United States; ^7^Department of Environmental & Occupational Health, University of Pittsburgh, Pittsburgh, PA, United States

**Keywords:** NTM, biofilm, CDC reactor, opportunistic pathogen, *Mycobacterium abscessus*

## Abstract

Antimicrobial silver materials for drinking water disinfection have become increasingly popular in building-wide systems (e.g., copper-silver ionization) and point-of-use applications (e.g., silver containing plumbing fixtures) to combat the microbial growth of drinking water associated pathogens that can cause infections in the immunocompromised (DWPIs). However, evaluations of various silver-containing treatments suggest that their efficacy is often temporary or incomplete. A potential explanation of these observations is insufficient dosing of silver into the drinking water to reduce these types of microorganisms, which are known to be more resistant to biocides. Instead, sublethal exposure may cause these microorganisms to adapt in ways that increase their resistance to disinfection. In this study, we assessed the effects of different silver concentrations on biofilms of clinically and environmentally isolated *Mycobacterium abscessus*, a biofilm-forming member of the drinking water microbiota with public health and environmental significance, in a bench-scale system operated to simulated the use patterns of antimicrobial showerheads. We found that high concentrations of silver significantly reduced biofilms cell densities and impacted cellular aggregation behavior, but ultimately made the resulting treated water non-potable due to the concentration of silver needed to solicit these effects. Silver concentrations that were more appropriate for drinking water applications resulting in limited reduction in viable *M. abscessus*. Additionally, transcriptomic analysis revealed that genes related to stress survival were upregulated in all experimental conditions: genes related to flavoprotein, chaperone, and protease synthesis, ribosome synthesis, and cysteine and methionine metabolism were upregulated in the lower dose condition, and peptidoglycan synthesis and antioxidant production were upregulated in the higher silver dose condition. These expressional changes may enhance survival and pathogenicity traits in *M. abscessus* after silver exposure. Overall, our findings indicate that silver exposure drives meaningful changes in biofilm behavior and gene expression in *M. abscessus* isolates, yet does not inactivate *M. abscessus* under the simulated conditions.

## Introduction

Silver as an antimicrobial agent in drinking water (DW) treatment has been suggested as a solution to combat the increase in infections caused by drinking water associated pathogens that can cause infections in the immunocompromised (DWPIs) (1, 2). These microorganisms can cause a range of infections, particularly respiratory infections in individuals with compromised immune systems (e.g., those at either age extreme or those living with immunosuppressing conditions). Treating these infections costs the United States healthcare system over $2.39 billion annually to treat (3). Among these, nontuberculous mycobacteria (NTM) are a major contributor to waterborne disease burden (2, 3), which is particularly challenging to address both clinically and environmentally due to their unique cell membrane (4) and penchant to form biofilms (5–7). Given these features there is a need for additional treatment to address these persistent microorganisms, since they are chlorine resistant and often thrive in biofilms in building plumbing (4, 8–10). Silver has been used since ancient times as a water disinfectant (11), and is thought to disrupt bacterial cell membranes, induce structural changes and functional impairment by interacting with DNA and proteins, and cause oxidative stress in bacteria by generating reactive oxygen species (ROS) (12, 13). Because of this, silver has been incorporated in both building-wide (14) and point of use DW treatment approaches (1, 15–17) to reduce DWPI proliferation. While both of these strategies report initial reductions in DWPIs (14, 18), these systems often return to pre-treatment microbial densities even when the silver treatment was still in use (17, 19–21).

Sub-lethal exposure to silver is a possible explanation to the recurrence of microorganisms, where the majority of the microbial density is reduced after initial exposure, but the dose is insufficient to eradicate all microorganisms present. Determining the optimal dose for DW is challenging due to the diverse microbiome (16, 22–24) with varying sensitivities to biocides. Furthermore, NTM in particular require higher biocide concentrations to achieve sufficient disinfection (10, 25), which is often attributed due to their unique waxy pseudo cell walls (4) in addition to other microbial survival mechanisms such as utilizing efflux pumps, enzymatic degradation of the biocide, utilization of alternate metabolic pathways, or cell membrane alterations (26, 27). It is also possible that sub-lethal exposure to silver may have unintended consequences on gene expression and microorganism characteristics, which could ultimately lead to water quality and public health implications such as antimicrobial resistance or increased pathogenicity. The purpose of this study was to assess the biocidal effect, characterize changes in gene expression and physical characteristics of biofilms containing *Mycobacterium abscessus* isolates, a NTM species common to DW with clinical relevancy (28), when exposed to varying concentrations of silver nitrate in a simulated antimicrobial showerhead environment. CDC biofilm reactors containing these biofilms were run with variable flows to mimic the hydraulic and water use patterns of a showerhead, and two different silver doses were introduced daily over the course of 1 week to simulate treatment during use. Biofilms were then recovered from the reactor and were assessed for abundance, biofilm formation behavior, and gene expression to determine the effects of silver exposure.

## Materials and methods

### Materials

CDC Biofilm Reactors (BioSurface Technologies Corporation, Bozeman, MT, United States) (29) were employed to assess the impact of different silver ion exposures (silver nitrate) conditions on the viability, biofilm structure, biofilm formation behavior, and gene expression of different isolates of *M. abscessus* grown on acrylonitrile butadiene styrene (ABS) coupons (BioSurface Technologies Corporation, Bozeman, MT, United States). *Mycobacterium abscessus* was assessed due to it being identified as a DWPI of major clinical (30) and DW biofilm (7, 31) relevance. Furthermore, the antimicrobial activity of silver ions were assessed against NTM due to it being much more resistant to CSI than other DWPIs such as *Legionella* (4, 32), hence effective inactivation against NTM would translate to easier to kill organisms. ABS coupons were used as this is the most common showerhead material used in manufacturing (33) and thus would be the most representative material as a biofilm substrate in this model system, and silver nitrate (Thermofisher, Waltham, MA, United States) was chosen as the silver ion source due to its known effects on microorganisms (11, 34) and precision of dosing in solution. Two experimental silver nitrate concentrations were tested: 48 mg/L and 480 mg/L Ag^+^ as silver nitrate. These values were chosen to represent the silver ion dose used in CSI treatment (32, 35) (48 mg/L) and an “extreme” treatment condition which was 10x the CSI dose used. Two strains of *M. abscessus* were used in this study: one strain, referred to from now on as the environmental *M. abscessus,* was isolated from a hot water system provided by the Special Pathogens Laboratory (Pittsburgh, PA, United States). The other *M. abscessus* strain, referred to from now on as the clinical *M. abscessus,* was isolated from a patient with an NTM lung infection and was provided by the DePas lab at the University of Pittsburgh (Pittsburgh, PA, United States).

### Initial characterization of *Mycobacterium abscessus* isolates, and silver nitrate fate and transport

Isolate sensitivity to silver nitrate was conducted in a series of microplate experiments. Both *M. abscessus* isolates were grown in liquid R2A media to 1.25 × 10^5^ cfu/mL as determined by plate counts on R2A agar, then transferred to a 96 well plate. Varying silver nitrate solutions (0 and 480 mg/L Ag^+^) were added to the wells and incubated at 37°C with gentle agitation for 10 min to produce contact times (CTs) of 0–4,800 mg/L min. After incubation, the plate was centrifuged at 5000 rpm for 2 min to pellet the cells, the supernatant was carefully removed, and the pellet was resuspended in fresh R2A media. The reduction in *M. abscessus* was determined via plate counts from the resuspended pellet. This methodology was repeated using silver nitrate concentrations of 0 mg/L and 48 mg/L and an incubation period of 100 min (achieving the same CT as the shorter, more concentrated trial) to determine if concentration or exposure time is the driving disinfection parameter (Supplementary Figure S1).

### *Mycobacterium abscessus* biofilm formation on coupons

Twenty ABS coupons (cleaned using 1% laboratory soap and water) per isolate (n = 40) alongside negative controls were incubated in 24-well tissue culture plates (Corning Incorporated, Corning, NY, United States) in the absence of silver, with one disk per well following the procedure outlined in (36). Briefly, each disk was covered with 5 mL of one of the two *M. abscessus* strains (grown to early stationary phase) and incubated for 72 h at 35°C with gentle shaking. Control disks were exposed to the same conditions as previously discussed except covered with 5 mL of growth media. At the end of the incubation time, loosely attached NTM were removed by dipping the coupon three times in diluted R2A media, and a final average coupon density of 5.1 × 10^6^ cfu/cm^2^ and 1.9 × 10^6^ cfu/cm^2^ was achieved for the clinical and environmental *M. abscessus* strains, respectively. Initial biofilm densities were determined via OD_600_ with reference to a previously developed standard of OD_600_ vs. viable NTM cell concentration of recovered biofilm and confirmed with additional characterization of five representative coupons for each isolate. Biofilms were grown without silver present in order to have a uniform initial exposure condition for characterization and to highlight the changes caused by silver during the reactor experiments.

### CDC biofilm reactor operation

Three CDC biofilm reactors each containing 20 ABS coupons were operated daily as follows for 7 days to simulate the shower environment which is composed of a short continuous flow phase (mimicking a showering event) followed by prolonged stagnation. Specifically, each day reactors were operated in continuous stir tank reactor (CSTR) mode for 10 min where the effluent flow rate was equal to the influent flow rate and the stir baffle was operating at 100 rpm. Influent for CSTR phase for each reactor was 0.45 μm (Thermofisher, Waltham, MA, United States) filtered shower water that was warmed to 40°C (the average showering temperature (37)) and contained either no silver nitrate (condition 1), 48 mg Ag^+^/L (condition 2), or 480 mg Ag^+^/L (condition 3) to achieve contact times of 0 mg Ag^+^/L*min, 480 mg Ag^+^/L*min, and 4,800 mg Ag^+^/L*min (Table 1). After 10 min of CSTR operation, each reactor was flushed for an additional 10 min (1 hydraulic retention time) with 0.45 μm filtered shower water to remove silver from the system and then allowed to stagnate for the remaining ~24 h with no stirring (in batch reactor mode). Silver ions were measured every day in the influent and in the effluent at the end of the 10-min exposure period via inductively coupled plasma mass spectrometry (PerkinElmer NexION 300 ICP-MS, PerkinElmer, Waltham, MA) analysis. After 7 days of operation all coupons were carefully removed for analysis with sterile forceps. These biofilms were then recovered and compared to the initial biofilm culture (biofilm grown on coupons and not put in a CDC reactor—condition 0 and condition 1).

**Table 1:** Summary of experimental reactor conditions.

Experimental condition	Reactor operation regimen	Silver dose (mg Ag^+^/L)
Condition 0	Not placed in reactor: served as initial biofilm characterization condition	0 (no silver exposure)
Condition 1	10 min in CSTR mode daily for 7 days	0 (no silver exposure)
Condition 2	10 min in CSTR mode daily for 7 days	48
Condition 3	10 min in CSTR mode daily for 7 days	480

Loss of silver due to adhesion to reactor components was assessed prior to conducting the final set of experiments and confirmed that the dose of silver nitrate administered in the influent was the dose that the biofilm was exposed to for the appropriate contact time.

### Characterization of *Mycobacterium abscessus* biofilms before and after silver exposure

The biofilm coupons retrieved from the CDC biofilm reactors after the 7-day experiment alongside extra coupons that had biofilms attached to them, but not placed in the reactors (referred to as “condition 0”) were assessed for biofilm density, silver accumulation, morphology characteristics, and biofilm kinetic behavior. One coupon per isolate per reactor was placed biofilm-side down in 4% para-formaldehyde for 4 h then visualized using microscopy at the DePas laboratory. Biofilm imaging and quantification was done by staining the fixed biomass using FilmTracer^™^ FM^™^ 1–43 Green Biofilm Cell Stain (Invitrogen, Waltham, MA, United States) and performing a 3×3 tile scan and simultaneous z stack. The biofilms from the remaining 4 coupons per isolate per reactor were recovered by gently rinsing with diluted R2A before being placed in a 50 mL microcentrifuge tube (Thermofisher, Waltham, MA, United States) along with a 1% Tween-diluted R2A solution and performing three rounds of 1-min sonication followed by 30s vortexing. Portions of the resulting biofilm suspension solution were used immediately for subsequent analysis.

Viability of culturable *M. abscessus* was assessed by performing plate counts on Middlebrook 7H11 media. ICP-MS analysis was performed to measure the concentration of silver within the biofilm, and the rest of the suspension was filter concentrated and extracted for RNA using the RNeasy Power Water kit (QIAGEN, Hilden, Germany), DNase-treated using the rigorous treatment of the TURBO DNA-free kit (Invitrogen, Waltham, MA, United States), then converted to cDNA using the iScript cDNA Synthesis kit (Bio-Rad, Hercules, CA, United States). Between each processing step, concentrations of the genetic material were obtained using the appropriate Qubit assay (Invitrogen, Waltham, MA, United States). The extracted RNA and cDNA were then stored at −80°C until absolute quantification of the *atpE* gene was assessed via droplet digital polymerase chain reaction (ddPCR; QX200, Bio-Rad, Hercules, CA) as previously described (1) (Supplementary Table S1).

After plate counts were finished, three representative colonies were chosen from each condition and used in aggregation assays to assess biofilm formation behavior as described in Spencer-Williams et al. (38). Briefly, *M. abscessus* isolates were grown in R2A liquid media after being taken from the plate, reinoculated, then allowed to grow for 35 h (38, 39). To assess difference in biofilm aggregation and disaggregation samples were taken at timepoints 0, 24, 33, and 35 h during the 35 h experiment by passing the culture through a 5 μm cell strainer (Pluriselect, Leipzig, Germany) and the optical density (OD_600_) of both the planktonic fraction (i.e., cells that passed through the strainer) and the aggregates (i.e., cells that remained on the strainer) were recorded. The OD_600_ value of the planktonic fraction was immediately recorded, while aggregates that collected on the strainer were resuspended in 6% Tween20 - PBS solution (Sigma-Aldrich, St. Louis, MO, United States). This suspension was then sonicated to resuspend remaining aggregates before recording the OD_600_ value. Both OD_600_ readings were used to calculate the planktonic to aggregate ratios.

### Transcriptomics

Extracted DNAse-treated RNA from both the clinical and environmental isolates were used to construct cDNA libraries using Illumina’s RNA-seq platform and sequenced on a NovaSeq 2000 by Argonne National Laboratory producing at least 5 million reads per transcriptome Quality control and data analysis of RNA sequencing data were performed on the Galaxy platform (40). Using Cutadapt version 4.8 (41), adapter sequences, sequences with a base quality of less than 20, sequences with a minimum length of less than 20, and Ns were removed from paired reads. Furthermore, the sequences from ribosomal RNA (rRNA) were filtered with SortMeRNA version 2.1b.6 (42) and curated rRNA databases in the Galaxy platform. After quality control steps, paired reads of all samples were pooled and *de novo* assembly was performed with Trinity version 2.15.1 (43). From the assembled transcriptomes, transcripts with a minimum transcript expression level of less than 1 were removed.

Differential gene expression analysis was performed with the estimated abundance of filtered transcripts based on Salmon (44) and DESeq2 (45). The homology annotations were performed on the filtered transcriptomes by Trinity, and predicted protein sequences of the filtered transcriptomes by TransDecoder version 5.5.0 (46), using the DIAMOND blastx mode, and blastp mode (47), respectively. The UniProtKB/Swiss-Prot (2023_03) (48), the curated database of the Galaxy platform, was used for a reference of the homology annotations. In addition, SignalP 6.0 h fast (49), HMMER version 3.4 (50) with Pfam database, and TMHMM 2.0 version 0.0.17 (51) were utilized to annotate the functional profiles of predicted protein sequences derived from de novo assembled transcriptomes. Finally, all annotations were collected and summarized using Trinotate version 3.2.2 (52), and the functional annotations were completed by additionally considering the Gene Ontology, DAVID (53), and STRING (54).

### Statistical analysis

All data was visualized and analyzed using R statistical software (Version 4.0.5). Significant differences (*p-*values < 0.05) between *M. abscessus* densities from the different silver exposure doses and aggregation behavior were assessed using paired Wilcoxan tests.

## Results

### Reduction of *Mycobacterium abscessus* biofilms was silver nitrate dose-dependent

**Figure 1:**
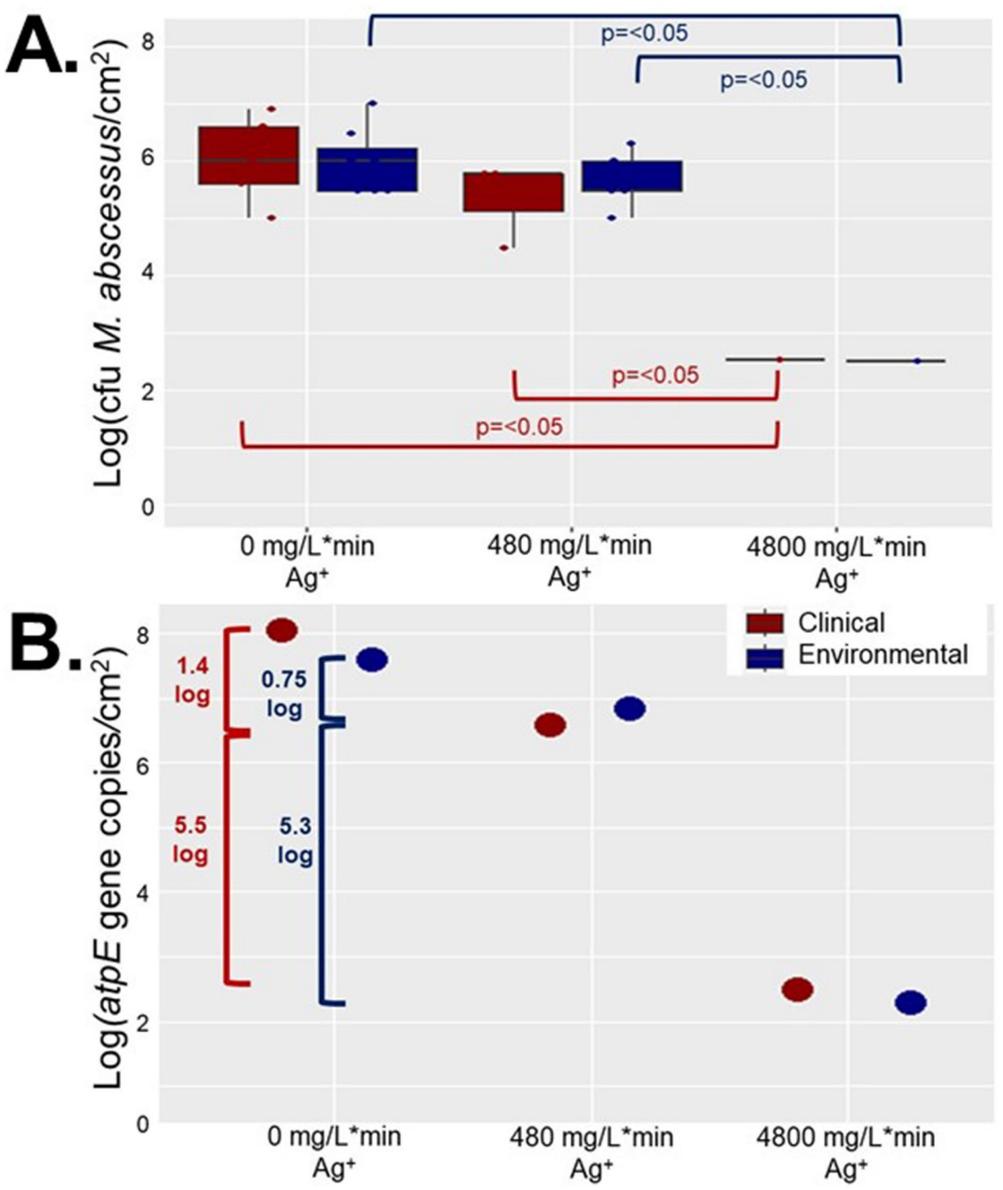
Recovered *Mycobacterium abscessus* biofilms after reactor operation for clinical (red) and environmental (blue) isolates quantified using **(A)**. plate counts (*n* = 8), and **(B)**. ddPCR results (*n* = 1). Significance in **(A)**. is denoted by brackets, but there were not enough samples to determine significance for **(B)**.

Operating CDC biofilm reactors simulating a showerhead environment containing ABS coupons seeded with an environmental or clinical *M. abscessus* biofilm the impacts of two different silver contact times (480 mg Ag^+^/L*min, and 4,800 mg Ag^+^/L*min) were explored. Biofilm density measurements from either *M. abscessus* strain recovered from coupons taken from the condition 1 reactor (0 mg Ag^+^/L*min) after 7 days of operation were not statistically different (*p* > 0.05) from measurements take from the coupons prior to being placed in the reactors (i.e., initial biofilm). Regardless of quantification method (microscopy, culture, or rt-ddPCR) there were no statistically significant reductions in *M. abscessus* biofilm densities between the control reactor and the 480 mg/L*min Ag^+^ reactor, meant to simulate the silver-only component of CSI (32, 35). However, there were decreases in both transcriptionally active abundances (6–6.9 log reduction) and culturable concentration (~3 log reduction) of *M. abscessus* in the 4,800 mg/L*min Ag^+^ reactor meant to operate as an extreme exposure scenario (Figure 1). Interestingly, there were no significant differences in the log reduction concentration between the clinical and environmental strains (Figure 1). These trends were also observed in the microscopy done on the biofilm (Supplementary Figure S2). It should however be noted that the order of magnitude decrease between the 480 mg/L*min Ag^+^ reactor and the 4,800 mg/L*min Ag^+^ reactor observed by viable absolute and culturing methods was not observed by microscopy, but this is likely due to dead cells remaining part of the biovolume adhered to the imaged coupons. Future studies employing microscopy should utilize live/dead staining or confocal microscopy to better characterize the living biovolume after silver exposure.

### *Mycobacterium abscessus* isolates displayed altered behavior after exposure to silver nitrate

**Figure 2:**
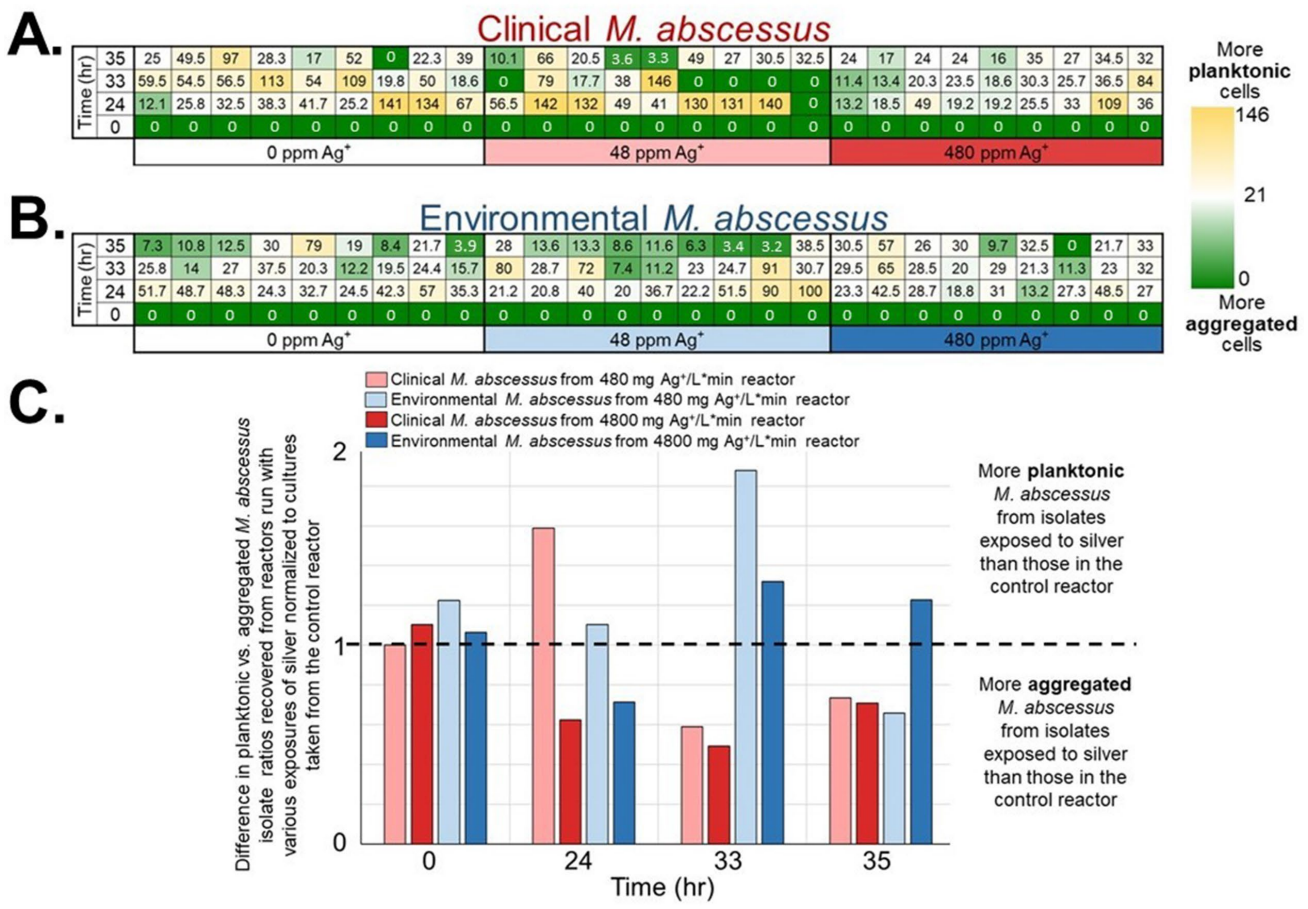
Planktonic vs. aggregate *M. abscessus* ratios (ranges in parentheses) for **(A)** clinical (0–163) and **(B)** environmental (0–100) isolates for the initial innoculates and after silver exposure each tile is one technical replicate (*n* = 9 per isolate × condition × time). Blue cells represent a smaller ratio, signifying a larger proportion of aggregated *M. abscessus* cells, while white/red cells represent a higher ratio (larger proportion of planktonic *M. abscessus* cells). The white cells represent the 50th percentile. The ratios were obtained by dividing the planktonic OD600 measurements by the aggregate OD600 measurements. **(C)** The values from **(A,B)** were normalized to the results of the isolates taken from the reactor with no added silver. Standard deviation was omitted for this figure to improve clarity.

Although significant inactivation was achieved in the high exposure reactor (4,800 mg/L*min Ag^+^) at least 1,600 *M. abscessus* cfu*/*cm^2^ remained after 7 days of chronic silver exposure which may have led to non-lethal effects such as behavioral modification, or changes in morphology which can have important consequences for public health. Aggregation assays conducted on isolates recovered from the reactors revealed that the aggregation behavior, or tendency for microorganisms to form a biofilm instead of being planktonic (dispersed in solution), changed with silver exposure (Figure 2). Based on ratios of planktonic to aggregated cells for the environmental and clinical *M. abscessus* isolates obtained from coupons not placed in the CDC bioreactors, peak dispersal occurred between 24 and 33 h, after which time they returned to a predominately biofilm phase (Supplementary Figure S3). Given that biofilm dispersal and formation behavior followed the same trends in the control reactor to those from coupons not placed in a reactor any observed differences in the silver reactors must be due to the different silver exposure conditions.

Overall, there were interesting differences in biofilm behavior between the strains and the different silver doses. A greater proportion of clinical *M. abscessus* strains isolated from the 480 mg/L*min Ag^+^ reactor entered a planktonic phase earlier than those isolated from the control reactor (peak disaggregation occurring at 24 h, instead of between 24 and 33 h in the control reactor; Figure 2). Interestingly, the environmental *M. abscessus* strains isolated from the lower dose silver reactor dispersed (entered a planktonic phase) later than those isolated from the control reactor (peak disaggregation occurring at 33 h, instead of between 24 and 33 h in the control reactor; Figure 2). Finally, the clinical *M. abscessus* strains isolated from the 4,800 mg/L*min Ag^+^ reactor exhibited very different biofilm behavior, as the majority of the community stayed in a biofilm / aggregated phase throughout the times sampled (Figure 2C).

### Gene expression was affected by silver nitrate exposure

**Figure 3:**
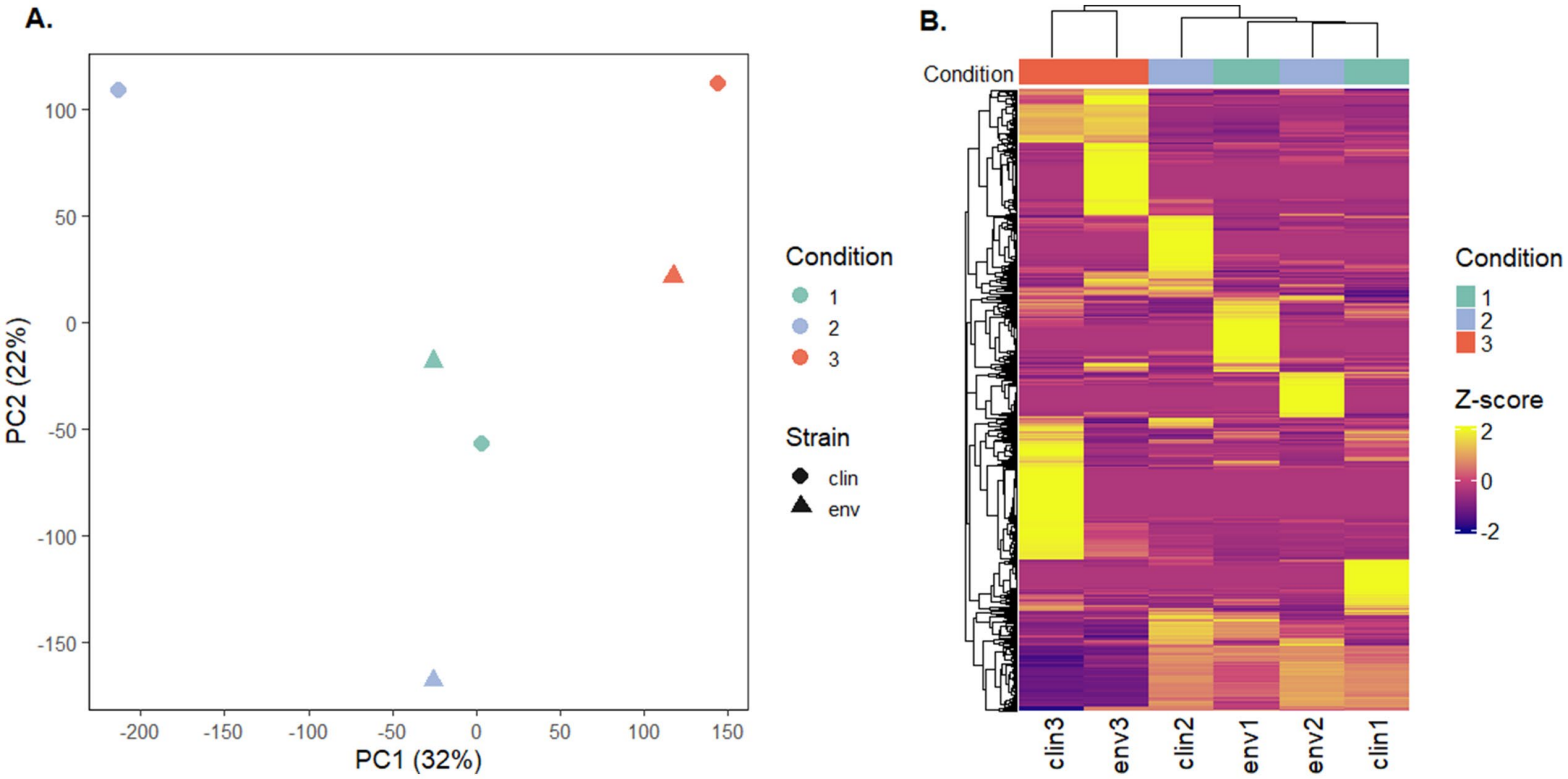
**(A)** Principal component analysis (PCA) of transcriptomic profiles, colored by experimental condition and shaped by strain type (clinical or environmental isolates). PC1 and PC2 explained 32 and 22% of the total variance, respectively. PERMANOVA indicated a clustering trend without statistically significant separation between groups (*p*-value = 0.2, R 2 = 0.554). **(B)** Heatmap of the top 1,000 most variable transcripts across all samples. Expression values are row-scaled (Z-score), and samples are hierarchically clustered. Columns are annotated by experimental condition.

**Figure 4:**
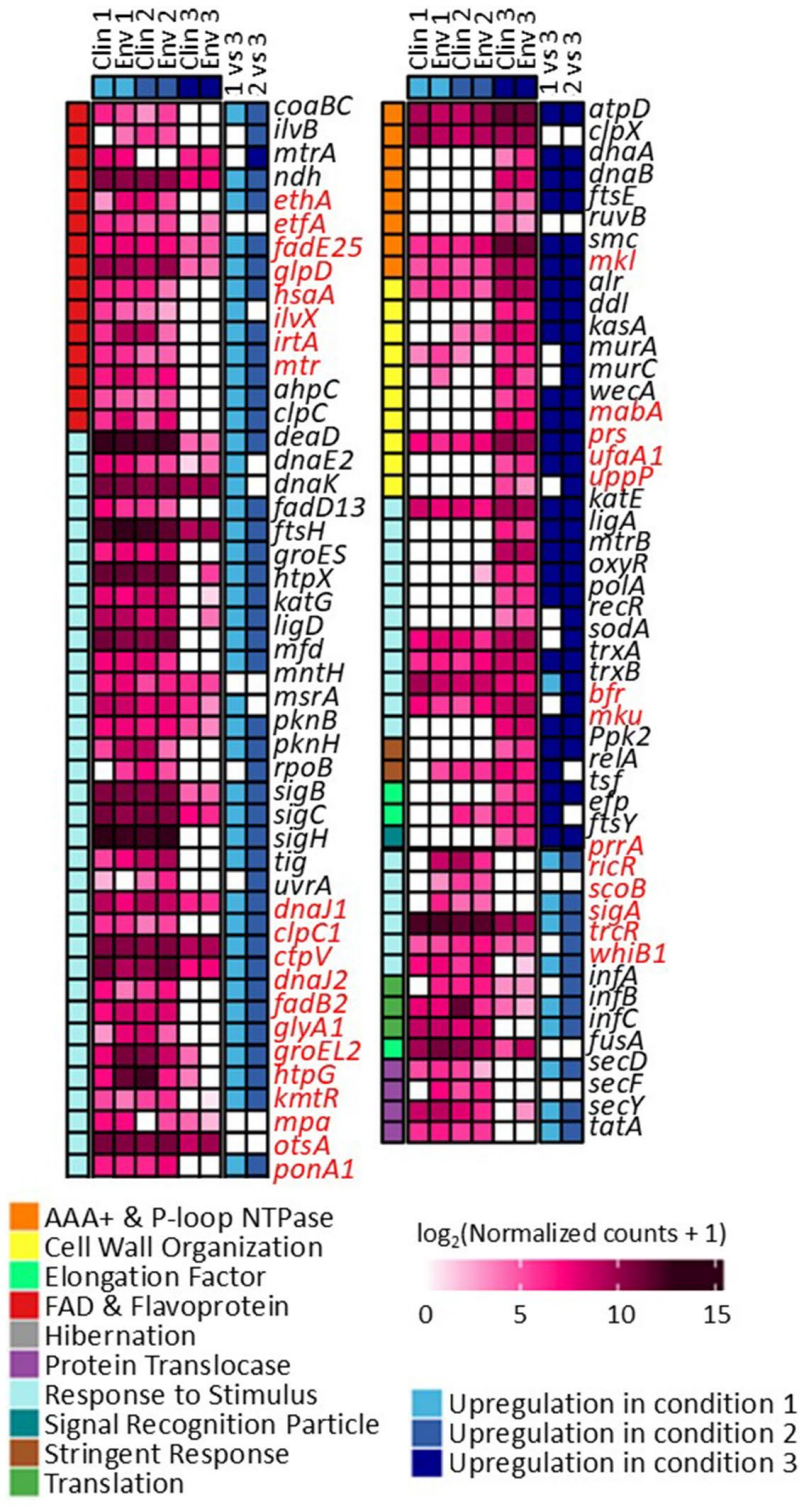
Differentially expressed genes and their functions in conditions 1, 2, and 3. Comparisons between each condition were based on adjusted *p*-value less than 0.05. Genes highlighted with red are not present in *M. abscessus*, but similar protein sequences have been reported in NCBI.

**Figure 5:**
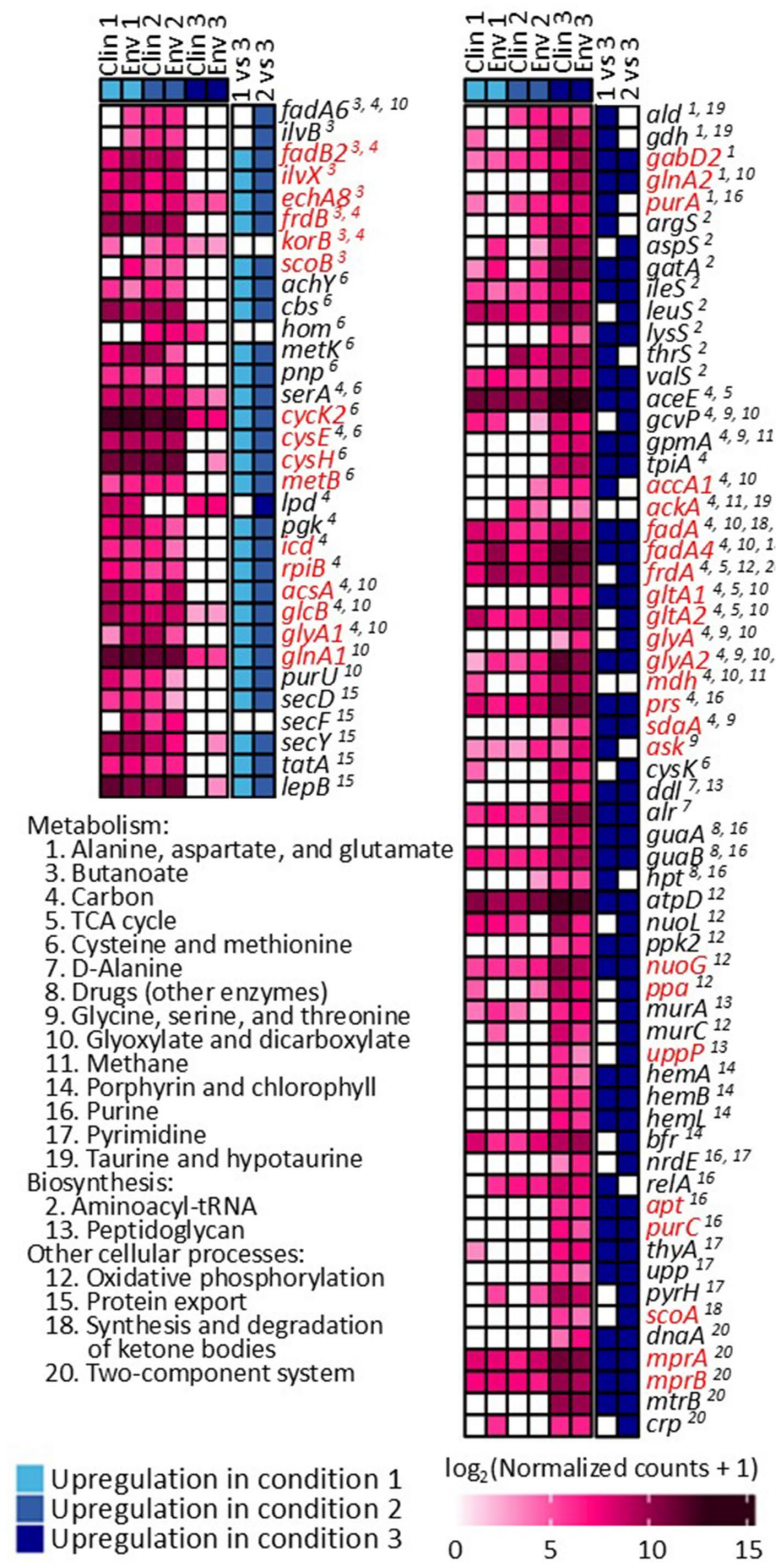
Differentially expressed genes and identified KEGG pathways in conditions 1, 2, and 3. Comparisons between each condition were based on adjusted p-value less than 0.05. Genes highlighted with red are not present in *M. abscessus*, but similar protein sequences have been reported in NCBI.

**Figure 6:**
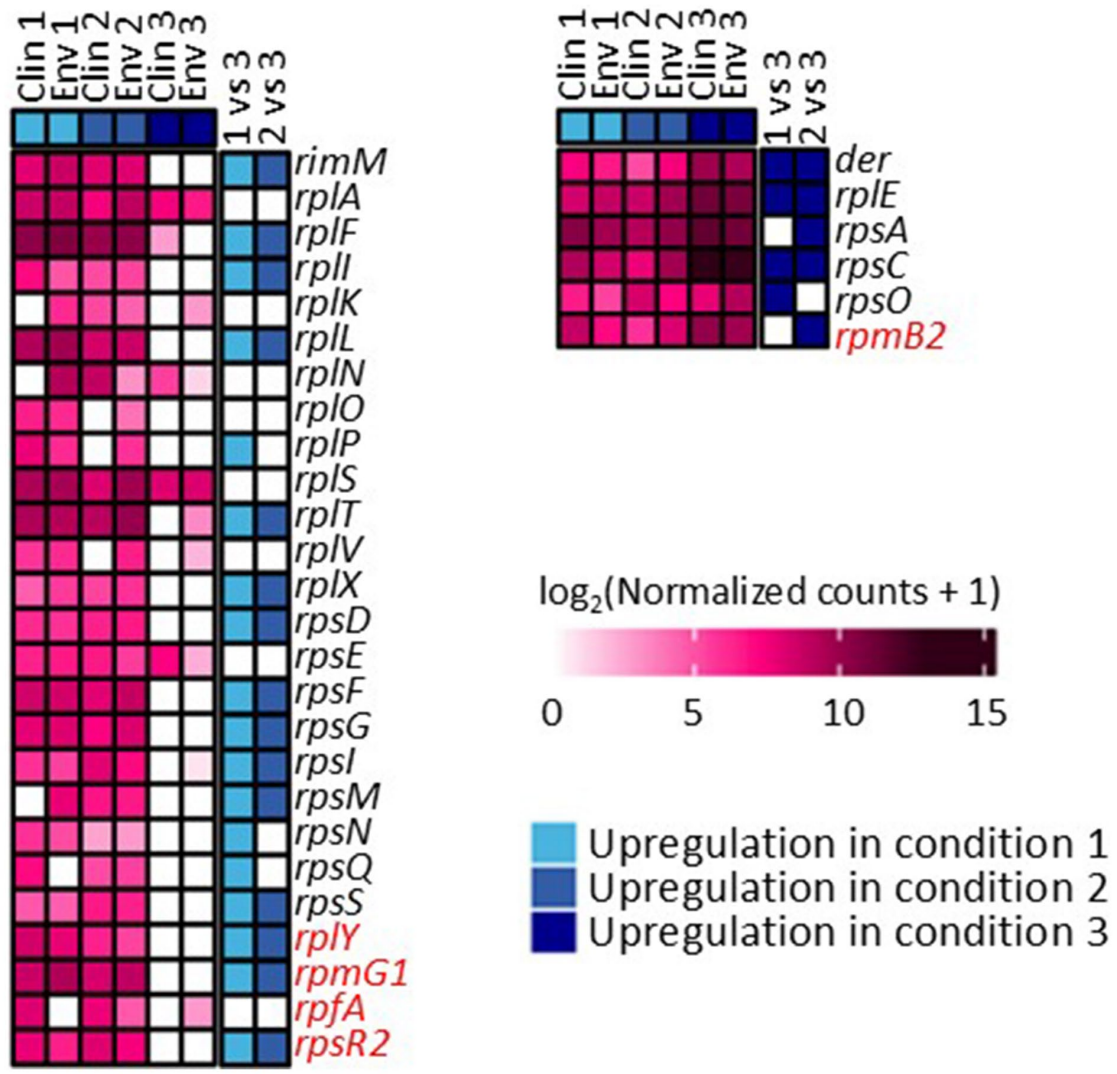
Differentially expressed genes associated with ribosome synthesis across conditions 1, 2, and 3. Comparisons between each condition were based on adjusted p-value less than 0.05. Genes highlighted with red are not present in *M. abscessus*, but similar protein sequences have been reported in NCBI.

The gene expression patterns under different levels of silver stress conditions suggest that *M. abscessus* has a condition-specific response to survive oxidative stress and starvation (Figure 3). It is interesting to note that there were no meaningful statistically significant differences between the clinical and environmental *M. abscessus* isolates regardless of silver exposure in addition to clear clustering observed on PCA analysis, which suggests that the isolates are transcriptomically similar to each other and experience similar changes in gene expression after exposure to silver (Figure 3A). The disparity between the differences observed in the aggregation studies and the similarity of the transcriptomic profile of the isolates may be influenced by epigenetic factors. While there is little literature that has explored the effects of silver on bacterial epigenetics, some exploratory studies have confirmed that oxidative stress triggers epigenetic response in a wide variety of bacteria (55, 107). Specifically, phosphorothioate internucleotide linkages have been identified *in vivo* as a response to oxidative stress (107). Interestingly, a study reported that *Salmonella enterica* displayed the same level of phosphorothioate activity after oxidant exposure as the pre-exposure condition due to the instability of these bonds during oxidative stress (108), which echoes the results reported in this work. Epigenetic responses to silver in bacterial model organisms appear diverse, with significant demethylation reported in *Escherichia coli* and methylation in *Staphylococcus aureus* (109). Ultimately, more work in the form of Enzyme-Linked Immunosorbent Assay-based measurements of DNA methylation levels must be done to determine if epigenetics are at play for these observed behavioral changes in *M. abscessus* due to silver exposure. Because of the lack of significant difference in gene expression between the clinical and environmental strains the data from both of these isolates were combined to increase sample size. A table summarizing the key differentially upregulated gene traits identified between experimental conditions can be found in Table 2. In culture prior to reactor inoculation, genes related to AAA + proteins, P-loop NTPases, and cell membrane formation and maintenance were more upregulated that those seen in *M. abscessus* isolates exposed to silver, which suggests that the upregulation of these pathways are important for survival in the absence of silver. Nutrient-limited conditions of the drinking water matrix in the control reactor (condition 1) and exposure to 480 mg/L * min Ag^+^ (condition 2) seemed to induce low and moderate stress to *M. abscessus*, respectively, as illustrated by changes in expression of flavin adenine dinucleotide (FAD) and flavoprotein genes, and stress-responsive genes, such as chaperones, proteases, genes involved in cell wall organization, peroxidase, and DNA repair (Table 2; Figure 4). Furthermore, in these conditions, ribosome biogenesis and cysteine and methionine metabolism were highly expressed compared to other conditions (Table 2; Figures 5, 6). The 4,800 mg/L * min Ag^+^ reactor condition caused severe stress on *M. abscessus*, resulting in the upregulation of genes associated with stringent responses, peptidoglycan and cell wall synthesis (Figure 4), and direct defense mechanisms such as DNA repair and antioxidants (Table 2; Figures 5, 6).

**Table 2:** Summary of key upregulated genes in reactor experiments.

Function	Genes most affected	Condition 1 (0 mg Ag^+^/L*min)	Condition 2 (480 mg Ag^+^/L*min)	Condition 3 (4,800 mg Ag^+^/L*min)
Cell wall organization and synthesis	*fadD13, pknB, pknH, alr, ddl, kasA, murA, murC,* and *wecA*	Upregulated	Upregulated	Upregulated
DNA repair	*uvrA, mfd*, *ligD, dnaA, dnaB, ruvB, ligA*, and *polA*	Upregulated	Upregulated	Upregulated
Stress Response	*MtrA*, *ndh*, *trxA, trxB, sodA, pnp, ahcY, metK, cbs, serA*, *trxA, trxB, soda, hom,* and *coaBC*	Upregulated	Upregulated	Upregulated (primarily oxidative stress response)
Translation factor	*infA, infB*, and *infC*	Upregulated	Upregulated	Not upregulated
Protein translocases	*secD, secF, secY*, and *tatA*	Upregulated	Upregulated	Not upregulated
Pathogenicity and infectivity	*sigC, dnaE2*	Upregulated	Upregulated	Not upregulated

## Discussion

### Silver nitrate is not a feasible drinking water disinfectant for *Mycobacterium abscessus* biofilms

Studies on the effects of silver have focused on biofilm prevention in laboratory conditions, using *Pseudomonas aeruginosa* as the model biofilm forming organism (56–59). *Mycobacterium* as a genus is known to be more resistant to silver than other DWPIs (17, 25, 32), and has been cited as a robust biofilm grower (4, 5, 7), which increases microorganisms’ resistance to antimicrobial substances (60). Therefore, it is unsurprising that *M. abscessus* biofilms survived regardless of silver ion dose or number of repeated exposures. The lack of significant differences between the control reactor biofilms and the 480 mg/L*min Ag^+^ reactor (Figure 1) may also be, in part, explained by the fundamental utilization of CSI, where this silver dose was modeled after. CSI is usually implemented in buildings where there is risk of DWPI infection, and is a technology where both copper and silver ions are being continually released into the DW conveyed in the building plumbing (14, 20), allowing for relatively long contact times to occur. Additionally, studies on the long-term efficacy of CSI show that initial treatment is often effective for combatting the target microorganism (often *L. pneumophila*), but regrowth occurs after the initial period of treatment (14, 18–20, 61). This study aimed to isolate the necessary silver ion concentration needed for an effective antimicrobial POU device, where the contact time is much shorter, and only silver is used, so it is possible that these differences caused less effective microbial inactivation. Bench-scale evaluations have demonstrated that there are additive antimicrobial effects when both copper and silver ions are present in a solution (62) so perhaps future antimicrobial silver showerheads should incorporate both ions. Further, there are a wide variety of different types of showerheads available on the market (63), and many that are marketed to be antimicrobial are constructed of materials other than ABS. Future studies should incorporate these other types of plumbing material as biofilm substrates in order to get a representative dataset of *M. abscessus* behavior in different showerhead material types.

**Figure 7:**
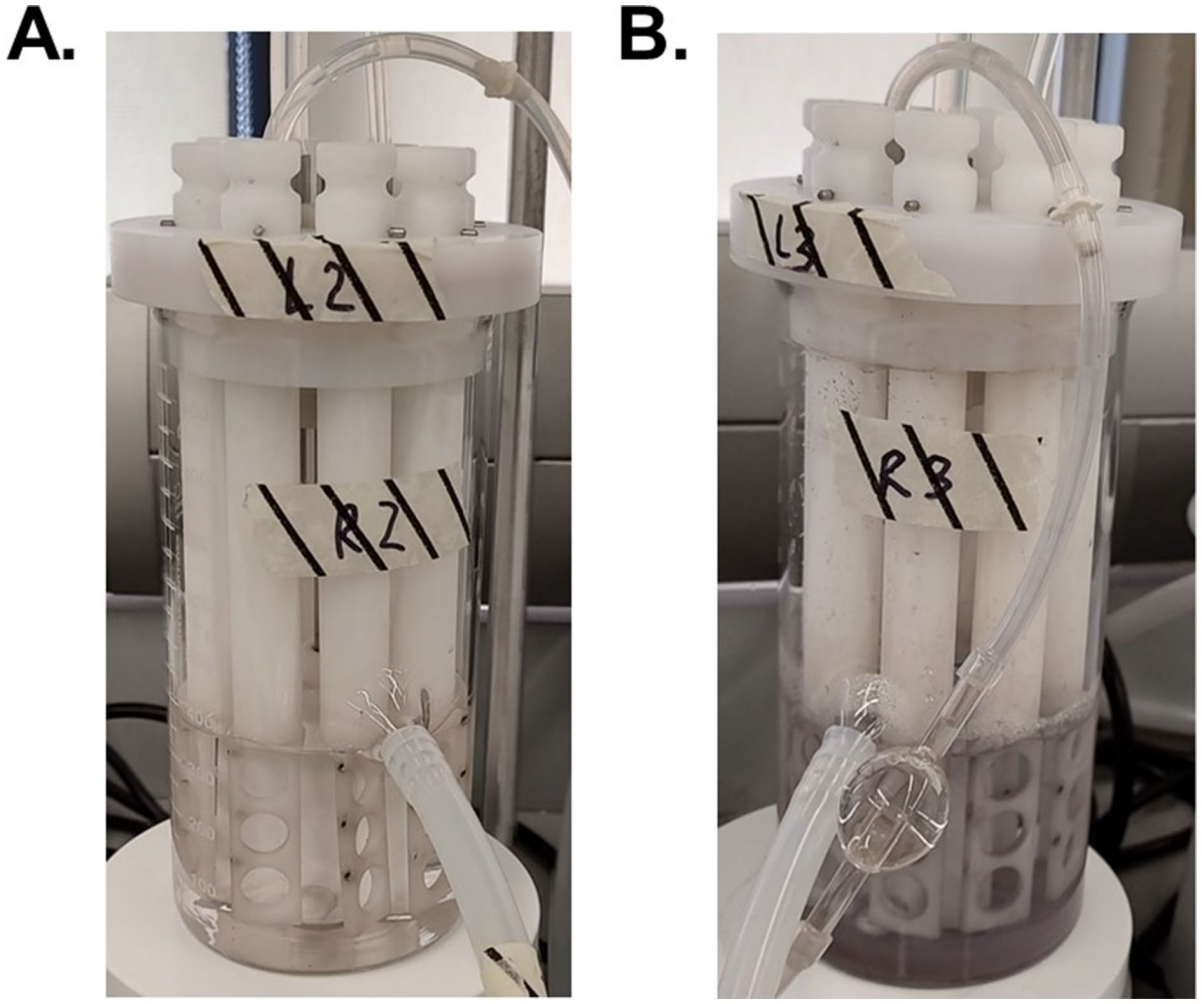
Visual discoloration cause by silver nitrate of the reactors with 480 mg Ag+/L*min **(A)** and 4,800 mg Ag+/L*min **(B)** after 7 days of reactor operation.

Although the 3 log decrease observed in culturable *M. abscessus* in the highest dosed reactors (4,800 mg/L*min Ag^+^) was significantly greater than reductions seen in the other two reactors (Figure 1), it is important to note that the concentration of silver nitrate that was added to the 4,800 mg/L*min Ag^+^ reactor was so high that it created DW that was no longer potable: there was significant discoloration of the feed and extensive visual silver deposition in all parts of the glassware used (Figure 7B). The national secondary drinking water standards state that silver must be under 0.1 mg/L (64), so other applications of biocide other than ionic silver in solution should be explored. This infeasibility suggests that silver ion exposure at this concentration is not appropriate for DW, and even then, the *M. abscessus* biofilm was not fully eliminated. There are a myriad of other silver administration strategies that have been patented for use in drinking water that span from silver-containing filter media to silver-releasing electrolysis, which report effective disinfection and suitability while maintaining lower aqueous silver residuals (65). A possible reason for this can be attributed to the much longer silver exposure time required for effectiveness: many of these technologies require multiple hours of contact with the silver-containing technology (65), and the current industry standard for testing antimicrobial materials (ISO 22196) prescribes a 24 h contact time before determining effectiveness (66). Results from this study corroborate this theory and determined that the driving parameter for disinfection was time, not silver concentration (Supplementary Figure S1). While this is important information for developing systems where long contact times are feasible, applications such as on-demand POU treatment. Based on these results, aqueous silver such as dissolved silver nitrate is not a reasonable antimicrobial for eradicating *M. abscessus* biofilms in situations where exposure only occurs for a short period of time like with POU applications. It is possible, however, that applications that have continuous silver exposure or increased time of silver exposure may be more effective at lower doses, but this condition was not tested in this study and should be further explored in future studies.

### Silver nitrate influences *Mycobacterium abscessus* biofilm behavior

Results obtained through the aggregation assay experiments are consistent with the biofilm work conducted in *Pseudomonas* (56, 67) as well as conventional knowledge about sublethal antimicrobial exposure: microorganisms contained in biofilms exposed to biocides tend to develop resistance to both the initial biocide, but also other antimicrobial substances due to the altered regulation of virulence proteins, quorum sensing, synthesis of polysaccharides, proteins essential for cellular adhesion and biofilm formation (56, 58, 68). Reasoning for the lack of disaggregation in the clinical isolate at the higher exposure condition (Figure 2) could be attributed to priming caused by previous *in-situ* survival from the human immune system and antibiotics, which may improve biofilm formation and robustness in this assay (28). Overall, these results suggest that clinical *M. abscessus* isolates which survive chronic repeated exposure to silver adapt to form a biofilm either as a defense or virulence mechanism similar to what is observed during active infection (30, 69, 70). Interestingly, no extracellular polymeric substances or quorum sensing related genes were found to be differentially expressed between these conditions based on log 2 fold change analysis, which was expected based on these traits’ importance in aggregation behavior. However, this may be an artifact of the analysis conducted since these genes may be expressed similarly across the experimental conditions. In line with our transcriptomic analysis described, which revealed elevated stress response gene expression in Condition 3, the aggregation behavior observed is consistent with a survival strategy commonly associated with biofilm formation under environmental stress. Biofilms provide enhanced resistance to biocides and other adverse conditions (5, 71), and the observed aggregation phenotype under silver exposure aligns with this known biofilm-associated adaptive advantage.

### *Mycobacterium abscessus* response to silver in the drinking water environment is dose dependent

It is known that non-ideal growth conditions such as low nutrient conditions and exposure to antimicrobial agents alter many microbial cellular functions, so it is no surprise that all three reactor conditions caused changes in the *M. abscessus* gene expression relating to stress response and survival. In the 0 mg Ag^+^/L*min and 480 mg Ag^+^/L*min reactors, many genes relating to DNA maintenance (e.g., *uvrA, mfd*, and *ligD*) (72), cell wall creation (e.g., *fadD13, pknB,* and *pknH*) (73, 74), and non-specific stress adaptation (e.g., *MtrA*, *ndh*, and *coaBC*) were upregulated (75–78). In the 4,800 mg Ag^+^/L*min reactor, essential survival genes for DNA repair (e.g., *dnaA, dnaB, ruvB, ligA*, and *polA*) (79), cell wall reinforcement (e.g., *alr, ddl, kasA, murA, murC,* and *wecA*) (80), and direct oxidative stress response (e.g., *trxA, trxB,* and *sodA*) (13, 81, 82) were upregulated (Figure 4). In these cases, it is possible that environmental conditions induced enough cellular stress to alter normal processes in the 0 mg Ag^+^/L*min and 480 mg Ag^+^/L*min reactors, but not enough to cause major expression shifts to survival states, whereas the *M. abscessus* biofilms recovered from the 4,800 mg Ag^+^/L*min reactor displayed significant shifts toward a more dormant survival state.

In other cases, some pathways were affected as a function of silver dose: there was increased gene expression related to protein synthesis of ribosomal large and small subunits, translation factors (*infA, infB*, and *infC*) (83), and protein translocase (*secD, secF, secY*, and *tatA*) in the 0 mg Ag^+^/L*min and 480 mg Ag^+^/L*min reactors, but ribosomal subunits, translocases (i.e., *secY*, and *tatA*), and translation initiation genes (*infA, infB*, and *infC*) were not upregulated in the 4,800 mg Ag^+^/L*min reactor (Figure 6) (83). Generally, bacteria inhibit ribosome biosynthesis under stress, entering a stationary phase or hibernation (83). Previous studies on *Mycobacterium* spp. have shown that ribosomal protein gene expression can be either suppressed (84–86, 110) or upregulated (87, 88) depending on the type and intensity of stress. The inhibition of ribosome synthesis in *Mycobacterium* spp. under stress is typically attributed to induction of stationary phase due to ppGpp accumulation (via *relA* gene expression), hibernation (via *hpf* gene and ribosome modulation factor), and entry into dormancy (via *dosR* gene expression) (82, 86, 89). However, there is limited information on the presence of genes other than *relA* in *M. abscessus* (Figure 4). Moreover, the relatively low expression of *relA* in the 0 mg Ag^+^/L*min reactor compared to other conditions suggests that ppGpp accumulation may not have reached levels sufficient to inhibit ribosome synthesis. Although *relA* is expressed in isolates taken from the 480 mg Ag^+^/L*min reactor, the expression patterns of other genes are similar to those in the silver-less reactor. Because of this, it is possible that *M. abscessus* increased ribosomal protein biosynthesis in isolates recovered from the 0 mg Ag^+^/L*min and 480 mg Ag^+^/L*min reactors as a rapid response mechanism to the imposed stress conditions, as reported elsewhere (90).

### Exposure to silver may prime *Mycobacterium abscessus* for enhanced survival and pathogenicity

*Mycobacterium* spp. treatment in clinical and engineered systems is often challenging due to innate properties of the genus (e.g., waxy pseudo-cell wall that is more difficult to lyse (4), flavin intensive cellular structure that protects from oxidative stress (78), ability to enter dormant states in periods of unfavorable conditions (91), among others), however exposure to biocides in non-lethal doses may further increase the microorganisms’ ability to develop resistance to biocides, or even promote characteristics that make them more infectious. It is known that sub-lethal exposure to antimicrobials encourage resistance formation (26, 92), and that for DWPIs such as *Legionella pneumophila*, environmental conditions in DW prime them to be more effective at infecting hosts (93–96). Understanding these links in microbial response between the DW and clinical environments may improve public health outcomes overall.

When exposed to silver, it is known that microorganisms combat this metal exposure through a variety of pathways. One such response is transporting the silver out of the cell via porins or efflux pumps (97, 98), which would indicate genes relating to these transport proteins would be upregulated in silver-containing conditions. Genes associated with AAA + proteins and P-loop NTPases were upregulated (Figure 4), both of which are essential for the operation of these efflux pumps, among other essential functions, which may indicate that the *M. abscessus* isolates were adapting to silver exposure through this method (99, 100). Additionally, antioxidant formation is often upregulated in microorganisms to combat reactive oxygen species generated by intracellular silver (62, 101), with many genes (e.g., *pnp, ahcY, metK, cbs, serA*, *trxA, trxB, soda,* and *hom*) associated with this upregulated in the silver-containing reactors (Figure 5), which also suggests that antioxidant formation is a major defense mechanism for *M. abscessus* during silver exposure (102, 103).

Other upregulated genes from the *M. abscessus* biofilms recovered from the 0 mg Ag^+^/L*min and 480 mg Ag^+^/L*min reactors have been linked to pathways associated with infectivity and pathogenicity in addition to stress survival in other *Mycobacterium* spp., with the transcriptional regulator *sigC* and DNA polymerase *dnaE2* specifically affected (Figure 4). There have been knock-out studies that have identified *sigC* as a critical part of disease severity in *M. tuberculosis* in both mice and guinea pigs, both of which reported similar viability *in-vitro* to the wild type, but significant decrease in virulence following infection (104, 105). This gene is upregulated during stress conditions to aid in the production of direct defense mechanisms during active lung infection, which exemplifies the link between environmental circumstances and potential clinical outcomes. Additionally, the increased expression of specific genes such as *dnaE2* in the less-lethal reactor conditions underscores the connection between environmental stress caused by the DW environment and under-dosed antimicrobial treatment and the cellular stress sustained during infection (Figure 4). Work also done on *M. tuberculosis* revealed that *dnaE2,* an extra gene copy coding for polymerase, is expressed in stress conditions and promotes mutagenesis in transcription as a mechanism for rapid adaptation to harsh environments (88). Boshoff et al. reported that *dnaE2* was upregulated after exposure to UV radiation, which yielded survivors that were more drug resistant than the initial culture due to the random mutagenesis after DNA damage. Additionally, knock-out strains lacking *dnaE2* revealed less disease persistence in murine models, suggesting that this gene plays a crucial role in the persistence and virulence of *M. tuberculosis* (88). Overall, the direct links between DW conditions serving as priming environments for infection have been extensively documented for other DWPIs such as *L. pneumophila* (96, 106), so it is possible that other stress conditions may create *M. abscessus* isolates that are more difficult to treat in hospital settings.

## Conclusion

Although silver can be an effective antimicrobial material in some circumstances, ultimately it is not the “silver bullet” for managing microorganisms of public health concern in DW contexts which have short residence times. In this study, we found that exposing *M. abscessus* biofilms to repeated silver doses to simulate a silver-containing POU shower fixture reduced densities by 3-logs at a silver concentration that rendered the treated water undrinkable. Additionally, sub-lethal silver exposure of either tested concentration caused changes in biofilm behavior (i.e., earlier biofilm dispersion or strong preference for remaining in a biofilm) compared to the control. Changes in biofilm behavior caused by silver exposure can have large impacts on the DW system and public health: earlier disaggregation as seen with clinical *M. abscessus* exposed to silver can spell higher likelihood of exposure of planktonic cells for consumers, whereas strong preference for biofilms in the case of the environmental isolate exposed to silver can lead to major sloughing events as the biofilm matures. Transcriptomic analysis revealed similarities between gene expression levels in the control reactor and the reactor dosed with 480 mg Ag^+^/L*min, where stress-responsive genes were upregulated, which is consistent to adaptation to the low-nutrient DW environment of the reactors. The lack of transcriptomic differences between these two conditions suggest that this dose of silver did not create more stress on these isolates, which was consistent with the viability, aggregation, and biofilm microscopy assay results in this study. However, significant differences in biofilm behavior and transcriptomic profiles were observed in the 4,800 mg Ag^+^/L*min reactor, suggesting that the biofilms were under severe stress by upregulating genes associated with direct defense mechanisms and peptidoglycan synthesis. Using silver at a concentration sufficient to combat biofilms was ultimately infeasible, and the lower concentration tested altered cell behavior and gene expression, which might suggest that using ionic silver alone in any concentration to treat DW with quick contact times could be inadvisable. While silver alone for a short residence time may not be effective, it is possible that silver could still be valuable for DWPI management in different DW applications, such as supplementing it with other antimicrobials like copper or increasing the exposure time to achieve similar contact times with lower silver concentrations. Future studies should explore these other approaches in addition to considering the potential changes in scaling up silver-containing systems to pilot or full-scale. Additionally, further genomic and transcriptomic characterization of microorganisms of concern like *M. abscessus* should be pursued in order to better understand how these microorganisms defend themselves against public health measures, and to search for better strategies to manage this ongoing water quality problem.

## Data Availability

The data that support the findings of this study are openly available in Zenodo at https://doi.org/10.5281/zenodo.14791314.
