# Supplementary material for: The effects of silver nitrate on Mycobacterium abscessus biofilms in a simulated antimicrobial showerhead environment
Source: Front Public Health. 2025 May 26;13:1572869. doi: 10.3389/fpubh.2025.1572869 (PMC12146310; doi:10.3389/fpubh.2025.1572869)
Supplement: Supplementary file 1 [file Data_Sheet_1.docx]

Supplementary Material

**The effects of ionic silver on *Mycobacterium abscessus* biofilms in a simulated antimicrobial showerhead environment**

Sarah Pitell^1^, Cheolwoon Woo^1^, Jill Millstone^2,3,4^, Janet Stout^1,5^, Leanne Gilbertson^6^, Sarah-Jane Haig*^1,7^

^1^Department of Civil and Environmental Engineering, University of Pittsburgh, Pennsylvania, USA

^2^Department of Chemistry, University of Pittsburgh, Pennsylvania, USA

^3^Department of Mechanical Engineering and Materials Science, University of Pittsburgh, Pittsburgh, Pennsylvania 15260, USA

^4^Department of Chemical and Petroleum Engineering, University of Pittsburgh, Pennsylvania, USA

^5^Special Pathogens Laboratory, Pittsburgh, Pennsylvania, USA

^6^Department of Civil and Environmental Engineering, Duke University, Durham, North Carolina, USA

^7^Department of Environmental & Occupational Health, University of Pittsburgh, Pennsylvania, USA

*** Correspondence:**Sarah-Jane Haig
sjhaig@pitt.edu

**Table S1**: Molecular primers, thresholds, and assay sensitivity for ddPCR analysis.

| Target | Forward (5'-3') | Reverse (5'-3') | Approx. Amplicon Size (bp) | Ref | Limit of Detection and Quantification (copies/20 µL) | Threshold |
| --- | --- | --- | --- | --- | --- | --- |
| *Nontuberculous mycobacteria* | FatpE | RatpE | 164 | 3 | 5.6 | 9800 |
| *atpE* gene | CGGYGCCGGTATCGGYGA | CGAAGACGAACARSGCCAT |  |  |  |  |
| Thermocycling Conditions | | | | | | |
| 95 °C for 5 min, [95 °C for 1 min, 59 °C for 1 min, 72 °C for 2 min] x 45, 4 °C for 5 min, 90 °C for 5 min | | | | | | |
| Reference: 1251 3. Radomski, N., et al., 2013. BMC Microbiol, 13(1), 277 | | | | | | |


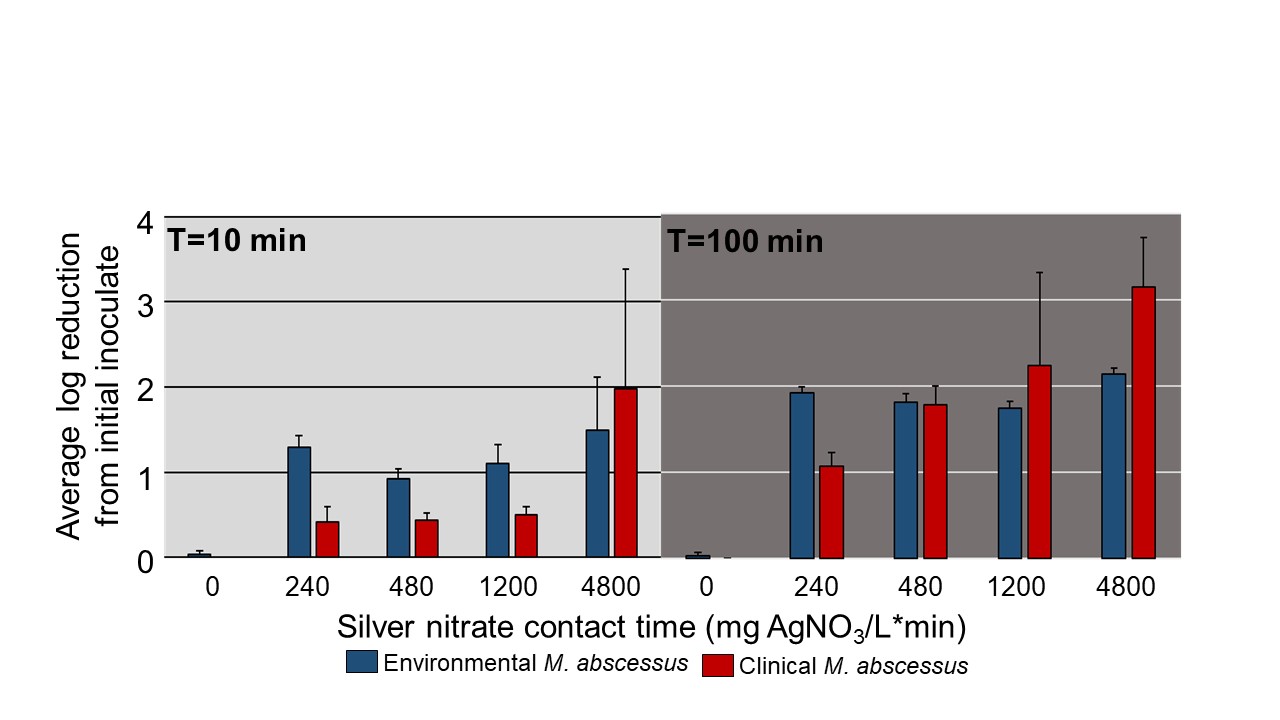


Figure S1: Average log reduction ± standard deviation of environmental (green) and clinical (orange) M. abscessus exposed to various silver nitrate concentrations for 10 minutes (light grey) or 100 minutes (dark grey). Each bar represents n=3 samples. The initial inoculate concentration for environmental and clinical isolates were 2.8 x 106 cfu/mL and 3.4 x 106 cfu/mL, respectively.


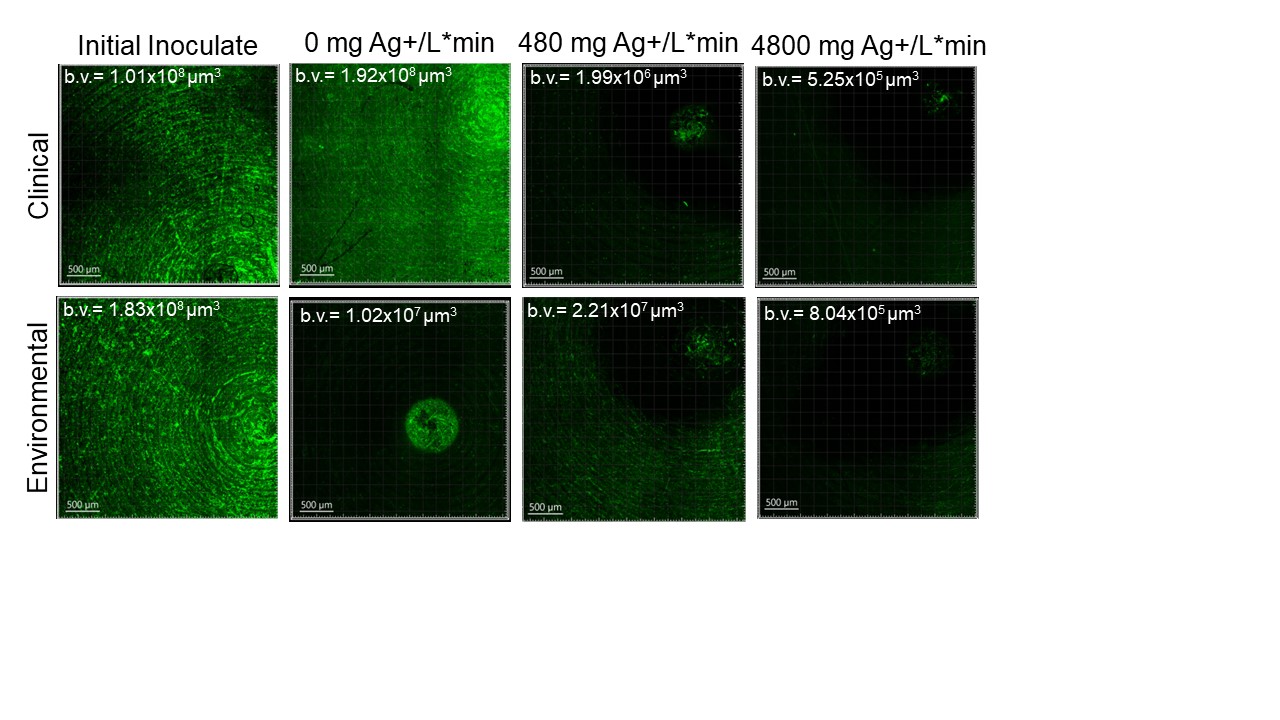


**Figure S2**: Microscopy results of *M. abscessus* biofilms recovered after 7 days of reactor operation by isolate (row) and exposure condition (column). Total biovolume (abbreviated b.v.) was included on each image that was quantified from zstack data.


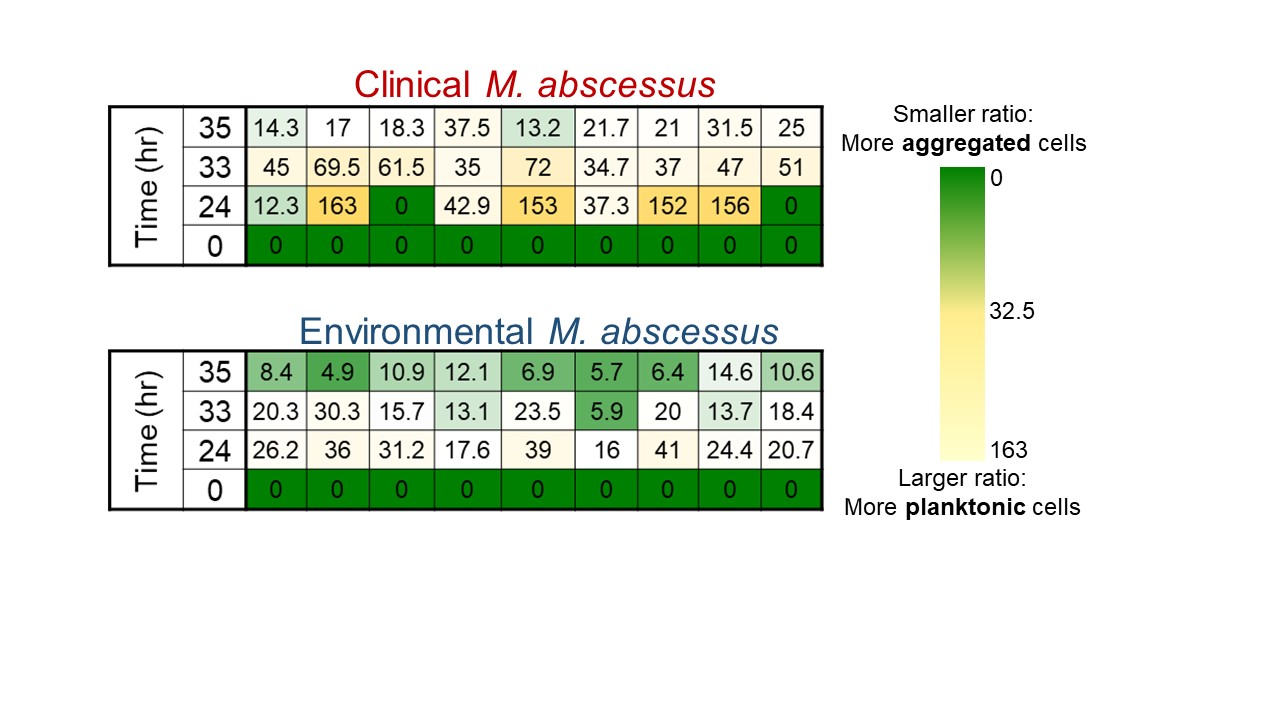


Figure S3: Planktonic vs. aggregate M. abscessus ratios (ranges in parentheses) for clinical (0–163) and B. environmental (0–41) isolates for the initial innoculates (each tile is one technical replicate, for a total of n = 9 per isolate × time). Green cells represent a smaller ratio, signifying a larger proportion of aggregated M. abscessus cells, while yellow cells represent a higher ratio, signifying a larger proportion of planktonic M. abscessus cells. The dark yellow cells represent the 50th percentile. The ratios were obtained by dividing the planktonic OD600 measurements by the aggregate OD600 measurements.
